# Visualization of the complete preprimosome reveals the structural mechanisms governing DNA replication restart

**DOI:** 10.1038/s41467-026-73239-1

**Published:** 2026-05-16

**Authors:** Peter L. Ducos, Alexander T. Duckworth, Kenneth A. Satyshur, James L. Keck, Timothy Grant

**Affiliations:** 1 John and Jeanne Rowe Center for Research in Virology, Morgridge Institute for Research, Madison, WI USA; 2 Department of Biochemistry, University of Wisconsin-Madison, Madison, WI USA; 3 Department of Biomolecular Chemistry, University of Wisconsin-Madison, Madison, WI USA

**Keywords:** Cryoelectron microscopy, Replisome, DNA-binding proteins, DNA replication

## Abstract

Replication restart pathways reinitiate DNA replication processes following their premature termination. In *Escherichia coli*, this essential process begins with regulated assembly of the preprimosome complex, comprising the PriA, PriB, and DnaT proteins, onto an abandoned replication fork. Here, we present two distinct preprimosome structures. One represents an intermediate stage in preprimosome assembly with a single DnaT C-terminal domain (DnaT^CTD^) bound to PriA/PriB/DNA. The second captures the mature preprimosome, in which filamentation of multiple DnaT^CTD^ molecules catalyzes the handoff of the single-stranded lagging-strand DNA from PriB to DnaT. The DnaT N-terminal domain forms a separate, independent oligomer in the mature structure. Taken together, our results detail the molecular mechanisms underlying replication restart initiation and regulation and suggest mechanistic similarities between DnaT and the canonical initiator protein DnaA.

## Abstract (web summary)

Restart of failed DNA replication processes is essential but poorly understood. Here, the authors present cryoEM structures of two preprimosome complexes, revealing how the PriA/PriB/DnaT complex assembles and matures. The findings suggest a possible DnaA-like mechanism for replisome recruitment.

## Introduction

In most bacteria, DNA replication begins at a single origin of replication (*oriC*) within a circular chromosome^1,2^. The initiator protein DnaA binds *oriC* in a sequence-specific manner, leading to oligomerization of the DnaA C-terminal domain (DnaA^CTD^) around DNA and double-stranded (ds) DNA melting^3–5^. The DnaA N-terminal domain (DnaA^NTD^) then self-associates and recruits the helicase/helicase loader (DnaB/DnaC in *Escherichia coli*), and DnaB is loaded onto the newly exposed single-stranded (ss) DNA^3,6–9^. Additional proteins interact with DnaB to form full replication complexes (replisomes)^10^ that drive bidirectional DNA synthesis^11^.

During replication, encounters with damaged DNA, transcription machinery, or other obstacles can cause the replisome to disengage prematurely, leaving an abandoned replication fork and a partially copied genome^12–15^. Unless the replisome is reloaded, such events are lethal in bacteria^16^. Specialized “replication restart” pathways have evolved to recognize abandoned replication forks in a sequence-independent manner and reload DnaB, reinitiating replication^17,18^. These pathways are also vital for processing displacement-loop structures produced by homologous recombinational DNA repair^18–24^.

**Fig. 1:**
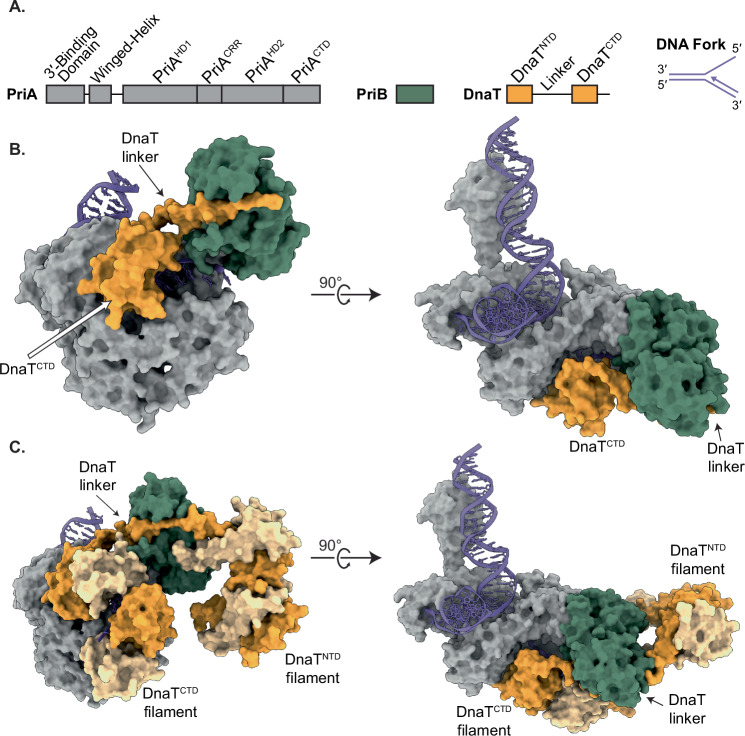
Cryo-EM analysis of the *E. coli* PriA/PriB/DnaT/replication fork complex. **A** Domain schematics of PriA, PriB, DnaT, and the DNA replication fork. **B**,** C** Molecular models of the intermediate (**B**) or mature (**C**) PriA/PriB/DnaT/DNA replication fork complexes. PriA (gray), PriB (green), and DnaT (dark and light orange) are shown in surface representation, while the replication fork (purple) is shown as a cartoon.

In *E. coli*, the major replication restart pathway is driven by the preprimosome protein complex composed of PriA, PriB, and DnaT. This complex identifies and remodels replication forks, recruits DnaB/DnaC, and directs DnaB loading onto lagging-strand ssDNA to resume DNA replication^17^. PriA initiates the process by binding to DNA in a structure-specific manner^25–30^. PriA is a DNA helicase with two RecA-like domains (PriA^HD1^ and PriA^HD2^) flanked by four additional domains (3′-binding domain, winged-helix domain, cysteine-rich region (PriA^CRR^), and C-terminal domain (PriA^CTD^)) (Fig. 1A). PriB, a homodimeric ssDNA-binding protein, is then recruited through direct interactions with PriA and lagging-strand ssDNA^31–33^. DnaT, which contains N- and C-terminal domains (DnaT^NTD^ and DnaT^CTD^) connected by a linker segment, is then added to complete the preprimosome. Several studies have suggested that DnaT could recruit DnaB/DnaC to the preprimosome complex and reload DnaB, although the mechanism remains unknown^34–40^.

Our recent cryogenic electron microscopy (cryo-EM) structure of the PriA/PriB/replication fork complex revealed the structural mechanism underlying the first steps of preprimosome complex formation^30^. PriA recognizes DNA replication forks via multiple contact points with the leading strand, lagging strand, and parental arms of the branched replication fork. Replication fork binding induces a domain rearrangement in which the PriA^CRR^ rotates ~80° from its position in apo PriA to form a pore that encircles the ssDNA lagging strand. This movement also exposes a surface on PriA^CRR^ onto which PriB docks, positioning PriB to bind lagging-strand ssDNA emerging from the PriA pore.

The structure of DnaT bound to the PriA/PriB/replication fork complex has not been resolved. DnaT is essential for DnaB loading onto replication forks in vitro, and deleting *dnaT* in *E. coli* causes severe phenotypes similar to those seen in *priA*-null strains^17,41–43^. DnaT association with the PriA/PriB complex requires DNA binding by PriA and is enhanced by PriB through the acidic DnaT linker binding to a basic groove on PriB^36,44^. PriB variants that disrupt the DnaT linker docking site are non-functional in *E. coli*^45,46^. Multiple DnaT molecules can assemble within the preprimosome, with higher-order DnaT oligomers forming as a function of DnaT concentration in vitro^44^, suggesting that DnaT oligomerizes within the preprimosome. Isolated DnaT^NTD^ and DnaT^CTD^ fragments self-associate in vitro, and sequence changes in the domains that impair DnaT oligomerization weaken DnaT ssDNA binding in vitro and block DnaT functions in vivo^37,41,47^. Thus, DnaT binding to the PriA/PriB/replication fork structure and self-association within the complex are essential for preprimosome activity.

To visualize assembly of the full preprimosome on replication forks, we analyzed the PriA/PriB/DnaT/replication fork complex via cryo-EM. Two distinct forms of the complex were observed. The first form includes a single DnaT^CTD^ and its linker docked onto the replication fork-bound PriA/PriB. The DnaT^CTD^ binds to a surface on PriA formed by the PriA^HD2^ and PriA^CTD^ domains that is occluded by the PriA^CRR^ in the apo PriA structure. Additionally, the DnaT linker element docks into a ssDNA-binding groove on PriB as predicted by prior observations^36,44^. The second form includes multiple DnaT^CTD^ molecules forming a helical filament that extends from the single DnaT^CTD^ observed in the first structure. DnaT^CTD^ filamentation is accompanied by a handoff of the ssDNA lagging strand from PriB to DnaT. The second form also contains density for a DnaT^NTD^ filament, which is observed projecting away from the remainder of the preprimosome. Based upon prior biochemical studies of DnaT, we posit that the monomeric and filamented DnaT preprimosome structures represent intermediate and mature forms of the complex. Intriguingly, the positions of DnaT domains in the mature preprimosome model reveal a DnaA-like arrangement where the DnaT^CTD^ filaments on ssDNA to mark the helicase-loading site and the DnaT^NTD^ oligomerizes to potentially recruit the DnaB/DnaC complex. This parallel suggests that replication restart and canonical replication processes may share significant mechanistic similarities.

## Results and discussion

### Cryo-EM structures of the *E. coli* PriA/PriB/DnaT/replication fork complex

DNA replication restart in *E. coli* begins with the assembly of a preprimosome complex, comprising PriA, PriB, and DnaT, onto an abandoned DNA replication fork^18,36,44^. Prior individual structures of PriA, PriB, DnaT^CTD^, and of the replication fork-bound PriA/PriB complex have provided important insights into the core preprimosome proteins and the physical basis underlying the first steps of their assembly into a complex^25,28,30–33,48–50^. However, the full PriA/PriB/DnaT preprimosome has not been visualized, leaving unanswered questions about the structural mechanisms that regulate preprimosome formation and the roles of DnaT within the complex. To address this gap, cryo-EM single-particle analysis was used to determine the structure of the complete *E. coli* PriA/PriB/DnaT/replication fork complex.

Purified PriA, PriB, and DnaT (1:2:3 molar ratio, see “methods”) were combined with a synthetic replication fork consisting of a 25-base pair dsDNA parental arm, a 15-base pair dsDNA leading strand, and a 15-base ssDNA lagging strand (Fig. 1A, Supplementary Fig. 1A, and Table 1). Complexes were isolated using size-exclusion chromatography and frozen onto EM grids. Cryo-EM analysis revealed two major structural classes, yielding high-resolution maps that were used to build two distinct preprimosome structures (Fig. 1, Table 2, and Supplementary Figs. 2, 3). The structures described herein represent ~50% of the final particles, and each contained PriA, PriB, DnaT, and DNA. Of the remaining particles, 6.8% contained PriA/PriB/DNA fork with no DnaT (representing our previously reported structure^30^), 1.1% contained two PriA molecules that shared a single DNA fork, and the remaining particles were classified into lower-quality versions of the reported structures with minor differences, such as small motions of the untethered DNA sections.

**Table 1:** Oligonucleotides used to construct the synthetic replication fork

Oligo name	Sequence (5′–3′)	Description	Source
oAD027	CGA GAC CGC AAT ACG GAT AAG GGC TGA GCA CGC CGA CGA A	Leading template strand	30
oAD028	GCC GCA GAC TCA TTT AGC CCT TAT CCG TAT TGC GGT CTC G	Lagging template strand	30
oAD029	TTC GTC GGC GTG CTC	Leading nascent strand	30

**Table 2:** Cryo-EM data acquisition and model statistics

Data collection and processing	Intermediate PriA/B/DnaT (EMD-74008, PDB-9ZBT)	Mature PriA/B/DnaT (EMD-74009, PDB-9ZBU)
Microscope	Krios G3	Krios G3
Voltage (kV)	300	300
Detector	K3 (Counting)	K3 (Counting)
Magnification (nominal/calibrated)	81,000	81,000
Data acquisition software	SerialEM	SerialEM
Electron exposure (e^−^/Å^2^)	100	100
Exposure rate (e^−^/pixel/s)	7.04	7.04
Number of frames per micrograph	102	102
Pixel size (Å)	1.085	1.085
Defocus range (μm)	−0.5 to −2.5	−0.5 to −2.5
Micrographs collected (no.)	1988 (0° tilt), 1999 (20° tilt)	1988 (0° tilt), 1999 (20° tilt)
**Reconstruction**		
Image-processing package	*cis*TEM	*cis*TEM
Total extracted particles (no.)	2,200,000	2,200,000
Final particles (no.)	222,000	212,000
Symmetry imposed	C1	C1
Resolution (Å)		
FSC 0.143 (unmasked / masked)	3.7/3.3	4.1/3.7
3DFSC Sphericity	.969	0.967
**Model composition**		
Protein Residues	1021	1483
Nucleotide Residues	77	80
Ligands	2	2
**Refinement**		
Refinement Package	Phenix	Phenix
CC (volume/mask)	0.75/0.74	0.73/0.73
Highest resolution used in refinement (Å)	4.2	4.6
R.m.s deviations		
Bond lengths (Å)	0.003	0.002
Bond angles (Å)	0.485	0.494
**Validation**		
Map-to-model FSC 0.5	3.7	4.0
Ramachandran plot		
Outliers	0	0
Allowed (%)	1.78	2.05
Favored (%)	98.22	97.95
MolProbity Score	0.95	1.10
Poor rotamers (%)	0	0
Clashscore (all atoms)	1.89	2.94
C-beta deviations (%)	0	0
CaBLAM outliers (%)	1.79	1.46

The first major structural class (25.04% of particles) yielded a 3.2-Å global resolution map that resolved a PriA monomer, a PriB dimer, a DnaT^CTD^ monomer and linker, and all arms of the DNA replication fork (Fig. 1B). Since DnaT oligomerizes within the preprimosome and self-association is critical for replication restart^37,41,44,47^, we refer to this form as an intermediate in the assembly of the preprimosome. The overall architecture of PriA and PriB within the structure was very similar to the previously solved PriA/PriB/replication fork complex structure, with the most notable difference being improved density for the PriA winged-helix domain, which has previously been shown to be flexible relative to the rest of PriA^28,30^. This improvement facilitated rigid-body fitting of the winged-helix domain but was insufficient for detailed molecular modeling. As described in greater detail below, a DnaT^CTD^ docks directly onto PriA. Lower resolution density was also observed for the DnaT linker, with none observed for the DnaT^NTD^ in the intermediate structure. The DnaT linker map resolution was insufficient to model the sequence with confidence. However, the map displayed continuous density with the DnaT^CTD^, and it clearly showed the linker bound to a groove on the PriB dimer.

The second major structural class (24.73% of particles) yielded a 3.6-Å global resolution map with density for a PriA monomer, a PriB dimer, and all arms of the DNA replication fork. A DnaT^CTD^ protomer was also included in the same location as that observed in the intermediate structure. The most striking addition in the second structure was the presence of multiple DnaT molecules, including density for a total of four DnaT^CTD^ and DnaT^NTD^ elements, and improved DnaT linker density (Fig. 1C). We posit that this structure represents the mature preprimosome.

The DnaT^CTD^ and DnaT^NTD^ oligomers within the mature preprimosome structure were homotypic, with DnaT^CTD^ and DnaT^NTD^ elements self-interacting and loosely connected by interdomain linker segments. The DnaT^CTD^ oligomer extended away from PriA as a left-handed helical filament. Fourteen of the 15 ssDNA lagging-strand bases were resolved in the structure, with eight residing in the DnaT^CTD^ filament interior. The DnaT^NTD^ density was weaker than that for the DnaT^CTD^, but it was sufficient to rigid-body fit two copies of an Alphafold3 predicted dimer structure of the domain^47^. Thus, a four-domain filament of the DnaT^NTD^ was modeled, although the density suggests a longer filament is possible. Continuous density connected one of the DnaT^NTD^ elements to the PriA-bound DnaT^CTD^ via the PriB-bound DnaT linker (Fig. 1C). Weaker density suggested similar connections between the remaining DnaT domains within the structure.

### DnaT docking onto PriA requires DNA-dependent PriA^CRR^ movement

**Fig. 2:**
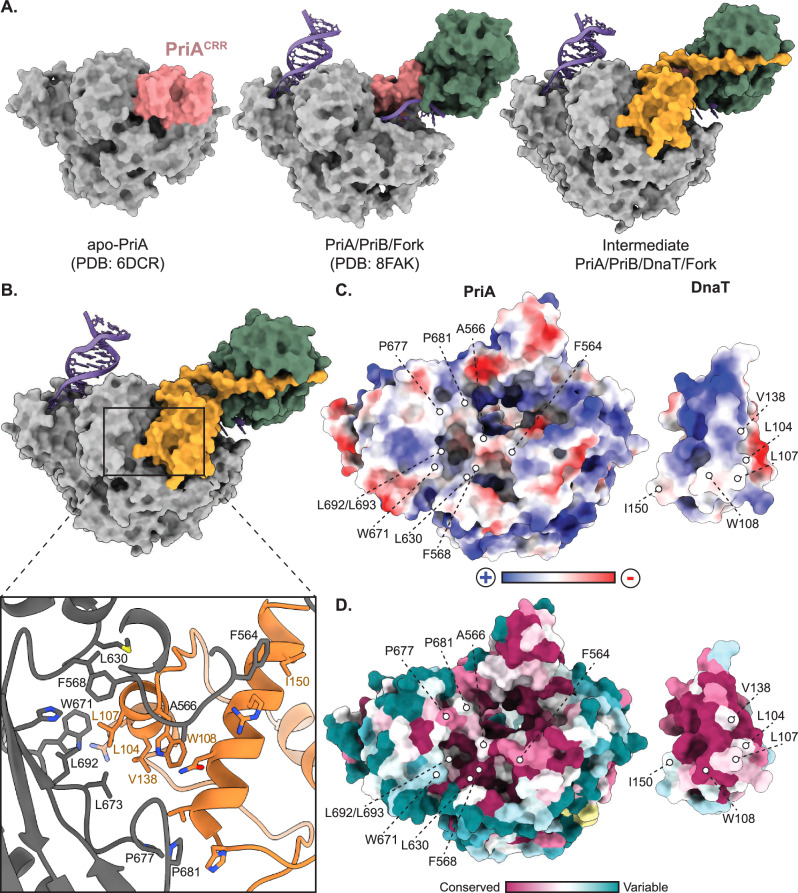
The DnaT^CTD^ interacts with the PriA^CTD^ via a hydrophobic interface. **A** Models of apo-PriA^28^, the PriA/PriB/replication fork complex^30^, and the intermediate PriA/PriB/DnaT/replication fork complex showing that the PriA^CRR^ (pink) occludes the DnaT (orange) binding site in the apo PriA structure. The color scheme for the other proteins is as described in Fig. 1. **B** Model of the intermediate PriA/PriB/DnaT/replication fork complex containing one DnaT^CTD^ molecule. The inset shows stick model representations of PriA/DnaT residues involved in interaction as assessed by the PISA server^51^. Hydrophobic residues are labeled. **C**,** D** Electrostatic potential (**C**) or conservation scores^52^ (**D**) mapped onto the PriA and DnaT binding regions. Key interface residues are highlighted.

In both preprimosome structures, a DnaT^CTD^ was observed docked onto a binding site that includes surfaces from PriA^HD2^ and PriA^CTD^ (Figs. 1, 2A). The PriA/DnaT interface buries a surface area of 640 Å^2^ (Fig. 2B, calculated via the PISA server^51^). The interface is highly hydrophobic (Fig. 2B, C) and is composed of residues that are evolutionarily well conserved (Fig. 2D, calculated via the Consurf server^52^, using bacterial species with an annotated *priB* gene). Binding at this position places the DnaT^CTD^ near the PriA pore that encircles lagging-strand ssDNA.

Interestingly, the DnaT-binding site on PriA is not surface-accessible in apo PriA due to the PriA^CRR^ position^30^, suggesting that the PriA^CRR^ must be repositioned for DnaT association (Fig. 2A). Relative to its position in apo PriA, the PriA^CRR^ is rotated ~80° to form the ssDNA pore within the preprimosome structures. The same structural change was observed in the PriA/PriB/DNA structure^30^, and is needed to expose the PriB binding site on the PriA^CRR^. PriA^CRR^ rearrangement, therefore, appears to serve as a structural switch that governs access of both PriB and DnaT to PriA, thus regulating preprimosome formation.

### The DnaT linker docks into a ssDNA-binding groove on PriB

**Fig. 3:**
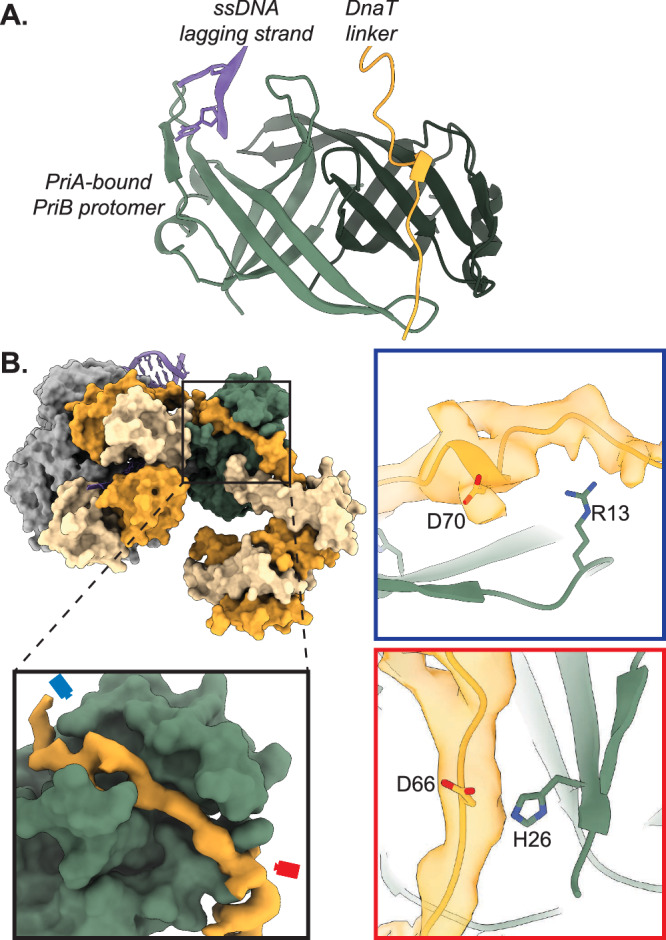
The DnaT linker docks onto a basic groove on PriB. **A** Model of the intermediate PriA/PriB/DnaT/replication fork complex showing the simultaneous binding of the ssDNA lagging strand (purple) and DnaT linker (orange) to the two PriB protomers (light/dark green). **B** Model of the mature PriA/PriB/DnaT/replication fork complex containing oligomerized DnaT. The inset shows PriB (green surface) and EM density for the DnaT linker (orange surface). Two views (blue and red boxes) show where the DnaT linker interacts with key PriB residues.

EM density for the linker that connects the DnaT^CTD^ and DnaT^NTD^ was observed in a groove on the surface of PriB in both preprimosome structures (Figs. 1, 3). The PriB homodimer has two basic channels, one in each protomer^31,53^. In the intermediate preprimosome structure (single-DnaT^CTD^), the lagging-strand ssDNA is bound to the PriB protomer that directly docks onto PriA, consistent with our prior PriA/PriB/DNA structure^30^, whereas the second basic groove is occupied by the DnaT linker (Fig. 3A). The dual-binding capability of PriB is supported by previous binding studies showing that DNA increases the affinity of PriB for PriA and that PriB increases the affinity of DnaT for the PriA/DNA complex^36^. The DnaT linker density is better defined in the mature preprimosome structure and, similarly, shows docking to a PriB protomer (Fig. 3B). However, as described in greater detail below, the lagging-strand ssDNA is no longer bound to PriB in the mature preprimosome structure but is instead in the interior of the DnaT filament.

Previous biochemical analysis has shown that PriB facilitates DnaT recruitment to the preprimosome^36,44^. Two features observed in the structures likely account for this effect: direct interaction between PriB and the DnaT linker and PriB stabilization of the PriA^CRR^ in a position that exposes the DnaT^CTD^ binding site. The DnaT linker is highly acidic, mimicking the negative charge of ssDNA, which facilitates binding to basic grooves on PriB^36,46,48^. PriB residues His26 and Arg13, and DnaT linker residues Asp66 and Asp70 have previously been shown to stabilize the PriB/DnaT interaction^36,46,48^. Due to the inherent heterogeneity of the linker, we could not precisely model the DnaT linker sequence. However, the main-chain model of this region suggests that Asp66 and Asp70 from DnaT are likely near PriB His26 and Arg13, respectively, potentially interacting with one another (Fig. 3B).

Although PriB is a homodimer with two potential binding sites for ssDNA or the DnaT linker, the function of this duality has remained unclear. PriB residues involved in binding the DnaT linker in the preprimosome partially overlap with those that interact with the PriA^CRR^ and ssDNA. This suggests that PriB is adapted so that one protomer binds to PriA and ssDNA while the second is available to bind the DnaT linker (Fig. 3A). This arrangement enables PriA and PriB to coordinate DnaT loading onto the ssDNA, thereby governing preprimosome maturation so that PriA and PriB binding to a replication fork precedes DnaT association. Such a mechanism could enhance the fidelity of preprimosome formation and DNA replication restart.

### The DnaT^CTD^ forms a helical filament around lagging-strand ssDNA

**Fig. 4:**
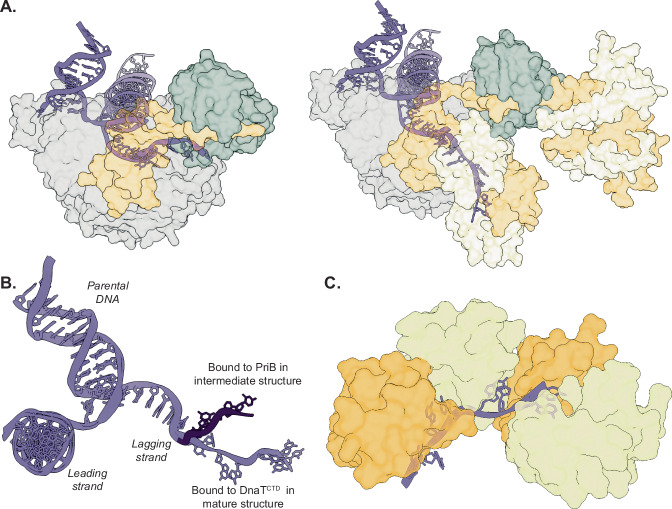
Lagging strand ssDNA is moved from PriB to the DnaT^CTD^ filament. **A** Transparent models of the intermediate (left) and mature (right) PriA/PriB/DnaT/replication fork structures depicting the different binding orientation of replication fork DNA. The color scheme is as described in Fig. 1. **B** Overlay of the replication forks from both structures. The ssDNA from the intermediate structure is colored dark purple to contrast its divergent path from the mature structure ssDNA. **C** Close up of the DnaT^CTD^ oligomer bound to DNA, showing two bases bound at the junction of each DnaT^CTD^ molecule.

The cryo-EM map of the mature preprimosome structure includes density for four DnaT^CTD^ elements. A prior crystal structure of *E. coli* DnaT^CTD^ bound to ssDNA^50^, which formed a helical filament in the crystal lattice, fit the protein density well (Fig. 1C). The densities corresponding to the second, third, and fourth DnaT^CTD^ molecules are progressively lower resolution, suggesting flexibility in the filament as it progresses away from the surface of PriA. The DnaT^CTD^ filament surrounds the ssDNA lagging strand (Fig. 4). The DnaT^CTD^/ssDNA filament shows a similar binding geometry to the previous crystal structure, with a nucleotide pair bound between each DnaT^CTD^/DnaT^CTD^ interface (Fig. 4C). Because the lagging strand remains bound to PriB when a single DnaT^CTD^ is present in the intermediate complex, our results suggest that DnaT filamentation is required to sequester ssDNA away from PriB. Together with prior biochemical analysis that defined a handoff where DNA is shuttled from PriB to DnaT^36,38,48^, and that DnaT^CTD^/DnaT^CTD^ interface residues are critical for oligomerization and replication restart^37,41^, the preprimosome structures suggest that DnaT linker docking and oligomerization are key for transitioning the lagging strand to DnaT.

The ssDNA in the mature preprimosome structure was oriented in the opposite polarity relative to the previous DnaT^CTD^/ssDNA crystal structure^50^. In the preprimosome, the DnaT^CTD^ filament was aligned in the 3′−5′ direction on the ssDNA, whereas in the crystal structure, the 5′−3′ direction was observed^50^ (Supplementary Fig. 1B, C). The reason for this difference is not known, but it could indicate that free DnaT^CTD^ favors ssDNA binding in the 5′−3′ direction. In the context of a replication fork, the directionality of DnaT filamentation is established by its binding to PriA and is thus restricted to 3′−5′ filamentation along the lagging strand ssDNA.

### The DnaT^NTD^ forms a multimer that is stabilized by PriB

**Fig. 5:**
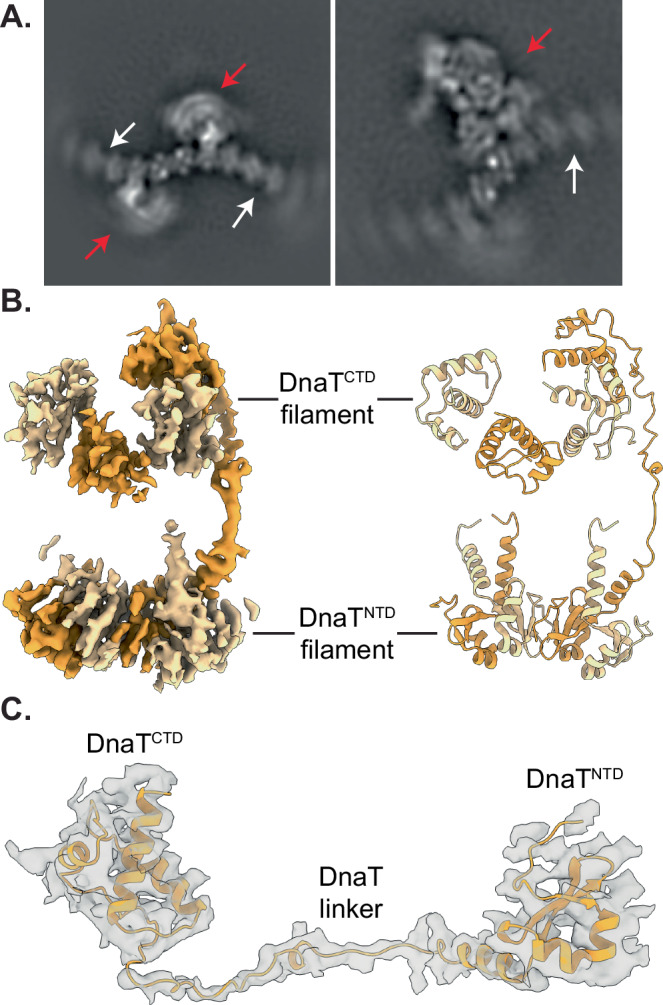
The DnaT^NTD^ forms an extended multimer. **A** 2D class averages from cryo-EM data processing showing extended DnaT^NTD^ oligomerization (white arrows) and PriA/PriB/replication fork complexes (red arrows). **B** Cryo-EM density (left) and cartoon model (right) of DnaT from the mature PriA/PriB/DnaT/replication fork structure. **C** Cryo-EM density of a full-length molecule of DnaT from the PriA/PriB/DnaT/replication fork structure.

Defining the DnaT^NTD^ structure and function has proven elusive. AlphaFold predictions suggest that the DnaT^NTD^ forms dimers, which can further oligomerize into extended filaments^47^. Mutation of key residues at the DnaT^NTD^ dimer interface (V10, F35) and at the dimer-dimer interface (F42, Y43) that impede oligomerization leads to defects in vivo, showing the importance of DnaT^NTD^ multimerization in replication restart^41,47,50^. Consistent with this model, several 2D class averages calculated from the EM data contain filamentous densities spanning large distances, often connecting multiple PriA/PriB assemblies (Fig. 5A). These superstructures of multiple PriA/PriB assemblies bound to long DnaT^NTD^ filaments are likely due to the high relative concentration of the purified proteins in the sample and not physiologically relevant, as only a single mature preprimosome would assemble per replication restart site. Portions of the filaments were also visible in the 3D reconstruction of the mature preprimosome, although the density was poorly resolved due to conformational flexibility and preferred orientation. Despite this, an AlphaFold3-predicted tetrameric DnaT^NTD^ (comprising two dimers) fit well as a rigid body into the density (Fig. 5B)^47,54^. Using the density for the DnaT linker, we connected the first DnaT^CTD^ subunit in the oligomer to a DnaT^NTD^, thereby resolving a full-length DnaT molecule within the complex (Fig. 5C).

The resulting structure revealed that the DnaT^CTD^ and DnaT^NTD^ form distinct oligomeric assemblies that coexist within the PriA/PriB/replication fork complex. In addition to its previously described interaction with the PriA^CRR^, PriB is positioned between these two oligomers, establishing contacts with both, as well as with at least one DnaT linker. In this manner, PriB serves as a structural scaffold that supports DnaT filament formation, suggesting a previously unrecognized role for PriB in organizing DnaT architecture during replication restart.

### Structural model of the DNA replication restart pathway

**Fig. 6:**
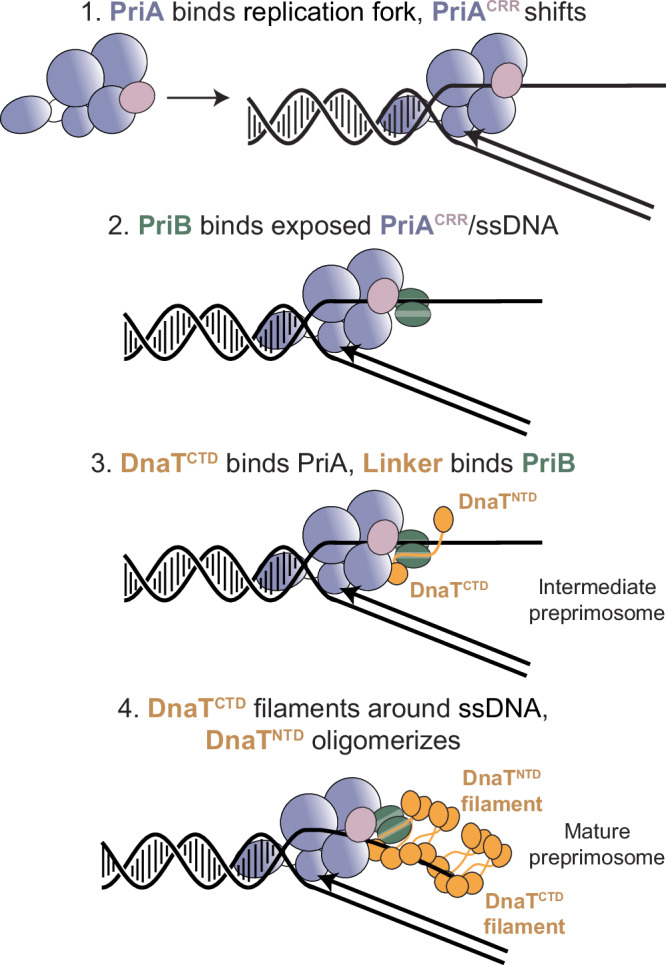
Model of PriA/PriB/DnaT preprimosome formation. PriA binding to a replication fork triggers rearrangement of the PriA^CRR^ to encircle lagging-strand ssDNA and exposes PriB and DnaT binding sites (1). PriB binds to the exposed binding site of the PriA^CRR^ and ssDNA (2). Once the PriA/PriB/replication fork complex is formed, a molecule of DnaT is recruited through interactions with PriA and PriB (3). Displacement of the ssDNA lagging strand from PriB occurs as additional molecules of DnaT are added, extending a DnaT^CTD^ filament around the ssDNA lagging strand. The DnaT^NTD^ oligomerizes and potentially recruits DnaB/DnaC (4).

Combined with prior structures of PriA^25,28^, PriB^31–33^, DnaT^CTD^^47,49,50^, and PriA/PriB/DNA^30^, the current structures complete a full description of preprimosome assembly on a DNA replication fork (Fig. 6). The process begins with structure-specific binding to a replication fork by PriA^25–29^. PriA remodels the fork to expose lagging-strand ssDNA. If the lagging strand is duplex, PriA helicase activity catalyzes remodeling; if it is ssDNA, PriA modulation of ssDNA-binding protein/ssDNA complexes assembled on the lagging strand is used^25^. Exposure of lagging-strand ssDNA triggers movement of the PriA^CRR^, allowing PriA to encircle the lagging strand. This movement simultaneously exposes the binding sites for PriB (on the PriA^CRR^) and DnaT (on the PriA^HD2^ and PriA^CTD^) to initiate formation of the preprimosome. The PriA^CRR^ movement thus represents the key step linking replication fork recognition to preprimosome complex formation. PriB docks onto PriA, while also interacting with the lagging strand^31–33^. The preprimosome structures presented here suggest a stepwise process for DnaT inclusion in the complex with a single DnaT monomer first binding to PriA/PriB/DNA, followed by additional DnaT molecules extending a filament away from PriA. Filamentation of the DnaT^CTD^ is accompanied by the ssDNA lagging strand moving from PriB to DnaT (Figs. 4, 5).

### Parallels between DnaT and DnaA suggest a conserved mechanism of helicase/loader recruitment for replication restart and canonical replication initiation

Following preprimosome assembly, DnaB/DnaC is recruited to the replication fork, and a DnaB hexamer is loaded around the lagging-strand ssDNA to reinitiate replication. Although this process has been reconstituted with purified proteins in vitro^43,44^, and DnaT has been suggested to serve as the DnaB/DnaC recruitment factor^34–36,38–40,50^, precisely how the preprimosome recruits DnaB/DnaC remains unknown. The preprimosome structures presented here have revealed a domain arrangement for DnaT that bears a striking resemblance to that of DnaA. Similar to DnaT, the DnaA^CTD^ forms filaments on DNA, and the DnaA^NTD^ extends away from the DnaA^CTD^/DNA complex^4,5,7–9^. Notably, while the DnaT^CTD^ forms a left-handed nucleoprotein filament, the DnaA^CTD^ filament is right-handed^4^. DnaA^NTD^ also forms homodimeric species, which can further associate into high-order oligomers that directly interact with DnaB/DnaC^7–9^. DnaA^NTD^ self-association is thought to provide a stabilized binding site for DnaB/DnaC that is not present with monomeric DnaA^9^. The similarities between DnaA and DnaT suggest that DnaT could potentially play analogous roles within the preprimosome. Consistent with this idea, previous studies have shown genetic interactions between *dnaT* and *dnaC* genes (present in the same operon), consistent with possible interaction between DnaT and DnaC or DnaB/DnaC^34,41,55^.

The DnaA/DnaT parallels could point to general features required to load replicative helicases in both replication initiation and replication restart. The first is a localization factor. For DnaA, this is accomplished through sequence-specific DNA binding to *oriC*, whereas for replication restart, localization is driven by structure-specific DNA binding by PriA. The second is DNA processing to create ssDNA for DnaB loading. For DnaA, processing arises from its intrinsic DNA unwinding activity and filamentation on ssDNA^3,5,8^, whereas for the preprimosome, processing can include lagging-strand unwinding by the PriA helicase or movement/exclusion of ssDNA-binding protein on the lagging strand by PriA, PriB and DnaT^26,28,56^. Finally, interaction with DnaB/DnaC is required along with DnaB ring loading onto ssDNA^50^. The similar domain arrangements between DnaA and DnaT suggest that DnaT^NTD^ could potentially play a direct role in DnaB/DnaC recruitment. The preprimosome structures described herein could be used to guide future in vitro and in vivo experiments to further define the interactions between DnaT and PriA/PriB and their role in initiating DNA replication. Future studies could also probe the intriguing parallels between DnaT and DnaA and potentially define the required characteristics of DNA initiation processes in bacteria.

## Methods

### Protein purification

*E. coli* PriA (N-terminal hexahistidine tag) and PriB were expressed and purified as described previously^30,57,58^.

BL21 (DE3) *E. coli* cells transformed with an overexpression plasmid harboring *E. coli* DnaT^36^ were grown shaking at 37 °C in Luria Broth containing 50 μg/mL each of kanamycin and chloramphenicol to an OD_600_ of ~0.4, then protein expression was induced by the addition of 1 mM isopropyl β-D-1-thiogalactopyranoside for three hours. Cells were pelleted and resuspended in 40 mL Lysis Buffer (50 mM Tris-HCl, pH 8, 10% glycerol, 10% sucrose, 100 mM NaCl, 5 mM ethylenediaminetetraacetic acid, 15 mM spermidine HCl, 1 mM phenylmethylsulphonyl fluoride, 1 mM benzamidine, 1 EDTA-free protease inhibitor tablet), lysed by sonication, and clarified by centrifugation. PolyminP was added to the supernatant over 30 min to a final concentration of 0.4% (v/v) on ice, the solution was allowed to precipitate for an additional 15 min and centrifuged to remove precipitates. Ammonium sulfate was added to the supernatant to a final concentration of 0.163 mg/mL over 30 min on ice, protein was allowed to precipitate for an additional 15 min and centrifuged to pellet DnaT. The DnaT-containing pellet was resuspended in Resuspension Buffer (10 mM Tris-HCl, pH 8, 10% glycerol, 500 mM NaCl, 2.5 mM ethylenediaminetetraacetic acid) and dialyzed overnight against QFF buffer (10 mM Tris-HCl, pH 8, 10% glycerol, 150 mM NaCl, 2.5 mM ethylenediaminetetraacetic acid). The resulting solution was filtered and loaded onto a HiPrep QFF column (GE) equilibrated with QFF buffer using an ÄKTA Pure FPLC system (GE) at 4 °C. The column was washed and eluted with a linear gradient of QFF buffer (150–1000 mM NaCl). Eluted protein was concentrated and loaded onto a HiPrep S100 column (GE) equilibrated in 575 mM NaCl QFF Buffer. Purified protein was concentrated, dialyzed overnight against storage buffer (10 mM Tris-HCl, pH 8, 50% glycerol, 150 mM NaCl, 2.5 mM ethylenediaminetetraacetic acid), and stored at −20 °C.

### Construction of synthetic DNA replication fork substrates

The synthetic DNA replication fork used in EM was generated as described previously (Table 1)^30^. The DNA lengths were chosen based on previous crosslinking and crystallography results. The PriA winged-helix domain contacts the parental duplex approximately 11–24 bp from the fork junction. We used a 25 bp parental duplex^28^. The PriA helicase domain contacts the lagging strand DNA approximately 5–6 nt from the fork junction^29^. This, along with PriB’s DNA binding footprint being around 10–15 nt, leads us to choose a 15 nt long ssDNA lagging strand length^31^. PriA only interacts with the 3’-most nucleotide of the leading strand DNA^28^. Thus, we chose the leading strand to also be 15 bp to maintain high efficiency annealing.

### Cryo-EM sample preparation and imaging

PriA (10 nmol), DNA replication fork (10 nmol), PriB (20 nmol, monomers), and DnaT (30 nmol, monomers) were co-incubated in 5 mL S200 Buffer (50 mM Tris-HCl, pH 8, 2 mM dithiothreitol, 5 mM ethylenediaminetetraacetic acid, 75 mM NaCl) on ice. The molar ratio for DnaT was determined under the assumption that it forms a trimer in solution as previously described^59,60^. The sample was centrifuged to remove aggregates and concentrated to 500 μL. The above order of addition prevented aggregation of DnaT. The complex was then purified on a Superdex 200 Increase 10/300 GL analytical size exclusion FPLC column (Cytiva) in S200 Buffer. Peak fractions were combined and concentrated to ~1 mg/mL. Samples were applied to Ultrafoil 1.2/1.3 grids (Quantifoil) that had been glow-discharged for 30 s using a GloQube Plus glow discharge system (Qurom Inc). The grids were plunge frozen at 4 °C using a Vitrobot Mark VI (ThermoFisher) under 100% humidity with a blot time of 4 s.

Movies were collected at 300 kV on a Titan Krios controlled by SerialEM^61^. 1988 movies werecollected using a Gatan K3 camera operating in CDS mode and BioQuantum energy filter with a 20-eV slit width at a calibrated pixel size of 1.085 Å. In a separate session, 1999 movies were collected using a Gatan K3 camera operating in CDS mode and BioQuantum energy filter with a 20-eV slit width at a calibrated pixel size of 1.085 Å at a 20-degree tilt. The datasets were collected with a total dose of 100 e/Å2 split into 102 fractions. Images were collected with a defocus range of 0.5 to 2.5 μm.

### Cryo-EM data processing

All 3987 movies were imported into *cis*TEM 2.065^62^ and processed using the standard workflow. Motion correction and contrast transfer function (CTF) estimation were followed by the exclusion of micrographs with CTF fits worse than 4.0 Å. Approximately 2.2 million particles were automatically picked using the disc picker and subjected to 2D classification, yielding ~857,000 particles in high-quality classes with clear high-resolution features. These classes showed significant structural heterogeneity. Comparison with previously published 2D class averages^30^ confirmed the consistent presence of PriA and PriB, often accompanied by additional variable densities.

To account for this heterogeneity, an ab initio 3D reconstruction was generated from the best 2D classes and refined using auto-refinement with C1 symmetry imposed. An initial 3D classification into five classes was insufficient to resolve the complexity of the sample, so a focused 3D classification was performed with six classes, focusing the classification around the PriB/DnaT region of the molecule. This enabled the isolation of distinct particle populations. Among these, the map with the best-resolved and highly populated classes corresponding to the PriA/PriB/DnaT/replication fork complex was selected and refined further. The resulting auto-refined 3D volume represented the intermediate preprimosome structure and accounted for 25.04% of the total particles (~212,000).

During previous rounds of refinement, diffuse density extending beyond the initial DnaT^CTD^ molecule was present in several classes, albeit at very low resolution. To further resolve this density, a 15-class 3D classification was performed on all ~857,000 particles. One class representing 14.06% of the particles showed more detailed density in this region. To increase the particle count in this class, a 1-round global classification of all particles was performed using this volume as a reference. This led to a volume with greatly improved features in this region. To further improve the detail, a 6-round focused 3D classification was performed, focusing on the region determined to be the DnaT filaments. One class representing 24.73% of the particles ( ~ 222,000) showed strong density for the filamentous DnaT regions. This was further auto-refined and resulted in a final 3D volume representing the mature preprimosome structure. A full overview of the processing workflow is presented in Supplementary Fig. 2.

Refinement of the intermediate preprimosome complex yielded a map with a global resolution of 3.2 Å (FSC 0.143), showing well-resolved density for PriA, PriB, DnaT, and DNA. As anticipated based on their flexibility, the extended DNA arms were unresolved in the final sharpened map. The oligomeric complex was refined to a resolution of 3.6 Å (FSC 0.143), clearly resolving PriA, PriB, DnaT, and DNA. The density corresponding to the DnaT^CTD^ oligomer was less well resolved beyond the first subunit in contact with PriA, suggesting conformational heterogeneity. While the remaining DnaT^CTD^ subunits were not sufficiently resolved for de novo model building, density for the overall oligomeric assembly remained interpretable.

The map also revealed additional features, including weak but traceable density for the DnaT linker binding to a PriB protomer. In the same oligomeric map, the first helix of the DnaT N-terminal domain was visible, while the rest of the domain and other DnaT^NTD^ subunits were not well resolved, likely due to a combination of preferred orientation and local heterogeneity. To improve the resolution of this region, focused classification was performed, yielding a subset of particles with enhanced density for the linker and proximal DnaT^NTD^, enabling further structural interpretation.

### Model building, refinement, and validation

Model building was carried out using UCSF ChimeraX^63^ and Coot^64^. For the intermediate preprimosome complex, rigid-body fitting was performed using previously solved structures of the *E. coli* PriA/PriB/replication fork complex (PDB: 8FAK)^30^ and a DnaT^CTD^ unit from the DnaT^CTD^/ssDNA crystal structure (PDB: 4OU7)^50^. For the mature preprimosome map, the full intermediate model (PDB: 9ZBT) was used as a template, and three additional DnaT^CTD^ subunits with bound ssDNA from the DnaT^CTD^/ssDNA crystal structure (PDB: 4OU7)^50^ were appended by rigid-body docking. For the DnaT^NTD^, an AlphaFold3-predicted^54^ hexameric model of the DnaT^NTD^ was rigid-body fit into the focused classification map. The DnaT linker region connecting the DnaT^CTD^ and DnaT^NTD^ was manually built.

All models were refined using Phenix real-space refinement^65^ and validated using Phenix tools. Refinement statistics are shown in Table 2. Model visualization and figure preparation were performed in ChimeraX and Coot. The final cryo-EM maps for the intermediate and mature PriA/PriB/DnaT/replication fork complexes were deposited in the Electron Microscopy Data Bank (accession codes EMD-74008 and EMD-74009), and the corresponding atomic models were deposited in the Protein Data Bank (accession codes 9ZBT and 9ZBU.

### Reporting summary

Further information on research design is available in the Nature Portfolio Reporting Summary linked to this article.

## Supplementary information

Supplementary Information

Reporting Summary

Transparent Peer Review file

## Data Availability

The cryo-EM maps for the intermediate and mature preprimosomes were deposited in the Electron Microscopy Data Bank (EMDB) with the accession codes EMD-74008 and EMD-74009, respectively. The final molecular models were deposited in the Protein Data Bank (PDB) with the identifiers 9ZBT and 9ZBU, respectively.
