# Supplementary material for: Visualization of the complete preprimosome reveals the structural mechanisms governing DNA replication restart
Source: Nat Commun. 2026 May 16;17:6489. doi: 10.1038/s41467-026-73239-1 (PMC13377028; doi:10.1038/s41467-026-73239-1)
Supplement: Supplementary file 1 — Supplementary Information [file 41467_2026_73239_MOESM1_ESM.pdf]

Visualization of the complete preprimosome reveals the structural mechanisms governing DNA replication restart

Authors

Peter L. Ducos[a,b,*], Alexander T. Duckworth[a,b,c,*], Kenneth A. Satyshur[c], James L. Keck[c,1], and Timothy Grant[a,b,1]

Affiliations

[a]John and Jeanne Rowe Center for Research in Virology, Morgridge Institute for Research, Madison, WI 53715, USA.
[b]Department of Biochemistry, University of Wisconsin-Madison, Madison, WI 53706, USA.
[c]Department of Biomolecular Chemistry, University of Wisconsin-Madison, Madison, WI 53706, USA.

*Peter L. Ducos and Alexander T. Duckworth contributed equally to this work.
[1]To whom correspondence may be addressed. Email: jlkeck@wisc.edu or tim.grant@wisc.edu

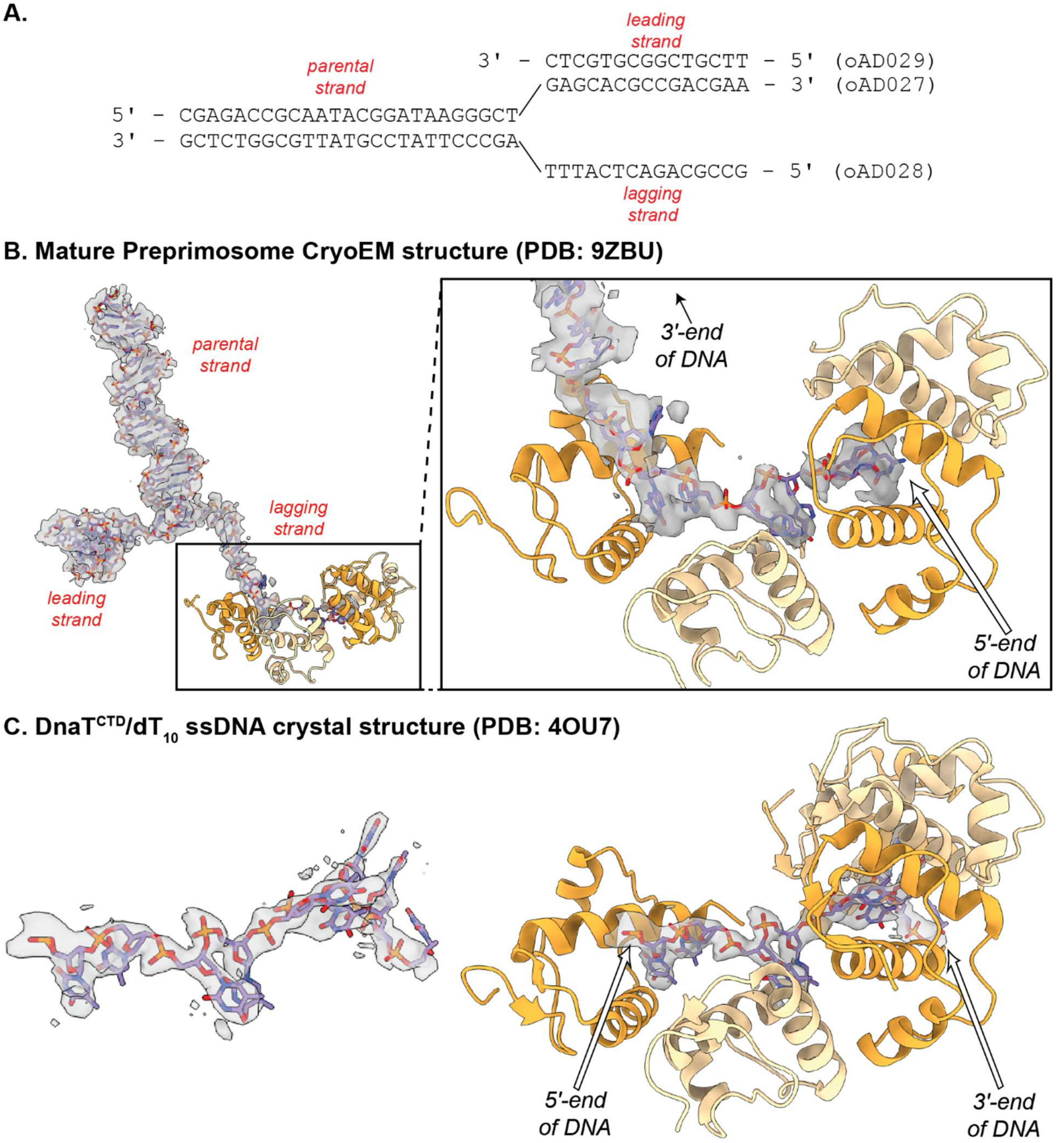

Supplementary Fig. 1. Comparing ssDNA polarity in DnaT^{CTD} filament structures.
A. Schematic of the synthetic DNA fork used for determination of the preprimosome structures. **B.** Atomic models of the DNA and DnaT^{CTD} filament in the mature preprimosome structure shown in stick and cartoon representations, respectively. The EM density for the replication fork is shown as a grey surface map. Inset – closeup of the DnaT^{CTD}/ssDNA filament showing the 5′-end of the lagging strand **C.** Atomic models of the DNA and DnaT^{CTD} filament from a previously solved crystal structure (PDB: 4OU7)[32] shown in stick and cartoon representations, respectively. The electron density for the ssDNA substrate is shown as a grey surface map.

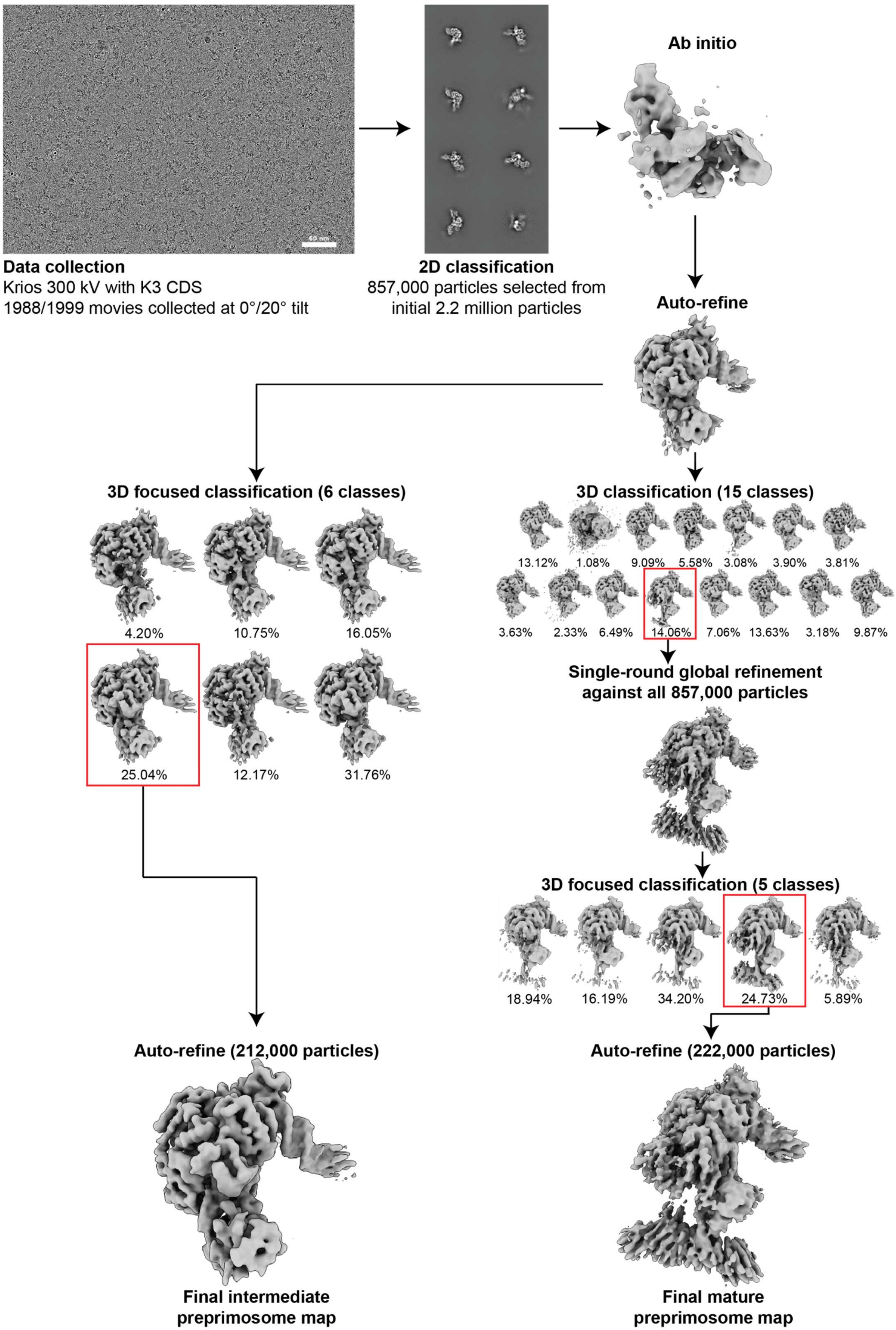

60 nm
Data collection
Krios 300 kV with K3 CDS
1988/1999 movies collected at 0°/20° tilt
2D classification
857,000 particles selected from
initial 2.2 million particles
Ab initio
Auto-refine
3D focused classification (6 classes)
4.20%
10.75%
16.05%
25.04%
12.17%
31.76%
3D classification (15 classes)
13.12%
1.08%
9.09%
5.58%
3.08%
3.90%
3.81%
3.63%
2.33%
6.49%
14.06%
7.06%
13.63%
3.18%
9.87%
Single-round global refinement
against all 857,000 particles
3D focused classification (5 classes)
18.94%
16.19%
34.20%
24.73%
5.89%
Auto-refine (212,000 particles)
Auto-refine (222,000 particles)
Final intermediate
preprimosome map
Final mature
preprimosome map

Supplementary Fig. 2. Cryo-EM data processing workflow.
Workflow for cryo-EM structure determination and resulting density maps. Representative cryo-EM micrographs from data collection and selected 2D class averages showing distinct particle views and conformations are shown. 3D reconstructions for all steps in the processing pipeline and particle counts are shown for the intermediate (left) and mature (right) preprimosome cryo-EM densities.

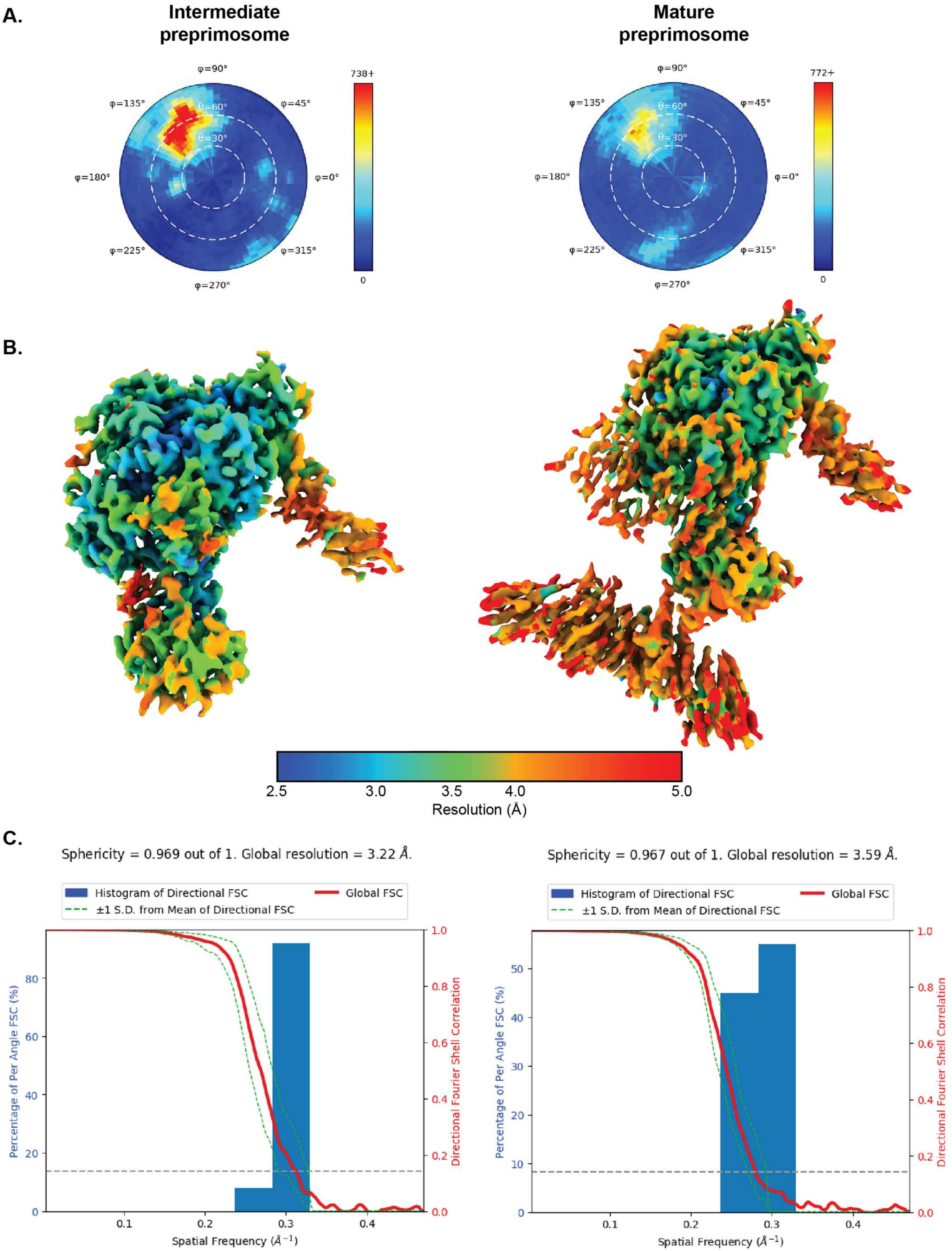

A.
Intermediate preprimosome
Mature preprimosome
φ=90°
φ=135°
φ=45°
φ=180°
φ=0°
φ=225°
φ=315°
φ=270°
θ=60°
θ=30°
738+
772+
0
B.
2.5
3.0
3.5
4.0
5.0
Resolution (Å)
C.
Sphericity = 0.969 out of 1. Global resolution = 3.22 Å.
Sphericity = 0.967 out of 1. Global resolution = 3.59 Å.
Histogram of Directional FSC
Global FSC
±1 S.D. from Mean of Directional FSC
Percentage of Per Angle FSC (%)
Directional Fourier Shell Correlation
Spatial Frequency (Å⁻¹)

Supplementary Fig. 3. Local resolution and 3D FSC plots.
A. Angular distribution plots illustrating particle orientation coverage for the intermediate (left) and mature (right) preprimosome cryo-EM densities. **B.** Local resolution maps of the intermediate (left) and mature (right) preprimosome cryo-EM densities color-coded by resolution from 2.5 to 5 Å. **C.** 3D Fourier Shell Correlation[48] (FSC) plots that illustrate map quality and resolution estimates for the intermediate (left) and mature (right) preprimosome densities.
