# Supplementary material for: Visualization of the complete preprimosome reveals the structural mechanisms governing DNA replication restart
Source: Nat Commun. 2026 May 16;17:6489. doi: 10.1038/s41467-026-73239-1 (PMC13377028; doi:10.1038/s41467-026-73239-1)
Supplement: Supplementary file 3 — Transparent Peer Review file [file 41467_2026_73239_MOESM3_ESM.pdf]

nature portfolio

Peer Review File

Visualization of the complete preprimosome reveals the structural mechanisms governing DNA replication restart

Corresponding Author: Dr Timothy Grant

This file contains all reviewer reports in order by version, followed by all author rebuttals in order by version.

Version 0:

Reviewer comments:

Reviewer #1

(Remarks to the Author)
In E. coli, the PriA, PriB and DnaT primosome restarts replication at abandoned DNA forks. In this manuscript, Ducos, Duckworth and colleagues report cryo-EM structures of the primosome. They identify two different assemblies on a stalled replication fork. One intermediate shows the recruitment of a single DnaT protomer. The second structure, which they describe as the mature primosome, contain multiple DnaT protomers arranged as a helical filament that wraps around single stranded DNA emanating from PriB. The authors note a loose structural similarity between DnaT and the bacterial replication initiator, DnaA. Multimeric protein filaments wrapping around single stranded DNA might play the same role during the ensuing helicase loading process, be it for replication initiation or fork restart. This is a nice piece of work that should be published after addressing the issues listed below.

1. Currently, the summary of the results constitutes ~35% of the Introduction. That could be shortened, in my opinion.
2. The authors claim that the presence of multiple DnaT molecules on PriA-PriB constitutes the structure of the mature replisome. I feel the authors should elaborate on why DnaT multimerisation is required for maturation, at this stage of the results section. Otherwise, such a statement belongs to the Discussion.
3. The authors claim that DnaT binds DNA with opposite polarity compared to the crystal structure. How did they determine polarity? Is the nucleic acid resolution sufficient to substantiate this claim? Is it continuous density between single stranded DNA and the duplex segment, where major and minor grooves can be discriminated? In either case, to substantiate their claim, the authors should add a supplementary figure, with the model overlaid to the transparent map. This is especially important given that the atomic model presented here has inverted DNA binding polarity compared to a previous co-crystal structure.
4. When drawing a parallel between DnaT and the DnaA initiator, the authors should point out that DnaA is a right-handed filament while DnaT is a left-handed filament and perhaps comment on the functional implications.
5. The figures could be improved by adding color code in every figure (space seems available) and pointing to specific features of note in the structure (e.g. ssDNA contact by PriB in Figure 1B for example). In Figure 1, I feel that purple DNA against highly shaded grey protein is barely visible, at least for me.
6. Figure 5. It would be useful for the authors to explain whether they think the DnaT filaments interspersed by PriB could be physiological or not. Also the figure could be improved. There are white arrows pointing at DnaT filaments but labels for PriB could be added.

Reviewer #2

(Remarks to the Author)
Ducos & Duckworth et al. used cryo-EM to determine structures of the E. coli primosome (PriA, PriB, DnaT), aiming to reveal the structural role of DnaT. They were able to determine two distinct forms of the complex, representing intermediate and mature primosomes. Based on these structures, they propose a structural model for the PriA/PriB/DnaT DNA replication restart pathway and a mechanism that shows parallels with replication initiation. The data are at high enough resolution to answer the questions.

This work will be of significance to the DNA replication and genome maintenance fields, with potential future use of this knowledge to target antimicrobial resistance.

Minor comments:

How were the synthetic replication fork lengths (25-base pair dsDNA parental arm, a 15-base pair dsDNA leading strand, and a 15-base ssDNA lagging strand) chosen?

The authors suggest that PriB is adapted so that one protomer binds to PriA and ssDNA while the second is available to bind the DnaT linker. Could the authors comment on the affinities of PriB with PriA, ssDNA, and DnaT, and whether those affinities would support such a mechanism?

In the cell, the ssDNA lagging strand would be bound by SSB (perhaps even RecA). The authors should comment on how the presence of SSB could alter their model, or how SSB is displaced by the DnaT filament.

Last paragraph of the introduction - the following sentence needs to be rewritten: Intriguingly, the positions of DnaT domains in the mature primosome model reveal an unexpected DnaA-like arrangement in which DnaTCTD filaments on ssDNA to mark the helicase-loading site, and with DnaTNTD element accessible for possible binding to DnaB/DnaC.

Figure 2 A, right – intermeditate = intermediate

Reviewer #3

(Remarks to the Author)
The manuscript by Ducos et al reports the structures of PriA-PriB-DnaT assembly on a model stalled fork structure. They have identified two forms of assembly, one the authors call intermediate and the other mature. The authors propose a stepwise process for DnaT association in the complex; a single DnaT monomer first binds to PriA/PriB/DNA, followed by additional DnaT (DnaT-NTD) molecules extending a filament through oligomerization. They propose that DnaTCTD forms filaments on the ssDNA lagging strand, replacing PriB with DnaT. Based on the model, authors propose the similarity of DnaB/C complex recruitment between DnaA-OriC and PriA-stalled fork systems, but the model has not been experimentally tested.

The detailed structure of the bacterial fork restart primosome is an important issue and the authors have been conducting the structural studies of the factors required for the primosome. The current study deals with a protein assembly required for the helicase loading and represents an important step for complete understanding of fork restart mechanism.

My comments are as follows.

1. Comment on the title: Visualization of the complete primosome reveals the structural mechanisms governing DNA replication restart

Authors misunderstand the term “Primosome”. Primosome can lay down primer RNAs for initiation of lagging strand synthesis while unwinding the duplex DNA with its helicase activity. Therefore, Primosome (for the fork restart) includes PriA, PriB, (PriC), DnaT as well as DnaB helicase (these are called preprimosomal proteins) plus DnaG primase.

Authors need to correct the description throughout the manuscript. The structure discussed in this manuscript is not Primosome. Preprimosome is Primosome minus DnaG (which is isolatable and is associated with helicase activity due to inclusion of DnaB helicase). The structure they studied in this manuscript could be called “prepreprimosome”.

2. It is of interest that two forms of assemblies were identified in the CryoEM. However, this does not show that the intermediate is a prerequisite for the assembly of more “mature" complex. This needs to be verified by examination of the structures during time course assembly of the PriA-PriB-DnaT-complex. Alternatively authors need to show the transition from the intermediate to mature form in biochemical assays of “prepreprimosome” assembly.

3. Authors need to verify the functional and biological significance of the structures identified.

3-1 They propose the critical roles of DnaTNTD oligomerization. To demonstrate this model, authors need to identify the residues that are important for DnaT-NTD ologimerization, mutate them and examine their effects on prepreprimosome assembly in vitro as well as on in vivo functions. Similar studies need to be conducted for the DnaT-CTD oligomerization interaction surface to examine the functional importance of DnaT-CTD filamentation.

3-2 Is the linker of DnaT that authors claim binds to PriB important for the function of DnaT? What is the function of DnaT(Δlinker) or DnaT mutant mutated in the PriB interacting amino acids (shown in Figure 3B) in the in vitro assays as well as in the cells?

Version 1:

Reviewer comments:

Reviewer #1

(Remarks to the Author)
The authors addressed all my concerns appropriately. I think this manuscript should be accepted for publication.

Reviewer #2

(Remarks to the Author)
My concerns have been addressed in the revisions.

Reviewer #3

(Remarks to the Author)
The authors have revised the manuscript and have substantially improved the text by incorporating relevant biochemical literatures on DnaT oligomerization and function. These additions strengthen the plausibility of the proposed model for preprimosome maturation and clarify the basis for interpreting the filamented DnaT state as a functionally relevant assembly.

The current revision relies primarily on previously published mutational analyses of DnaT NTD and CTD oligomerization, rather than testing predictions that arise directly from the present structures.

Both DnaTNTD and DnaTCTD can self oligomeiize, and the authors state that oligomerization of the both domains is important. The mutations that inactivate the oligomerization activity of DnaT are reported to lose its functions.

However, I would like to emphasize that my original concern was not simply whether DnaT oligomerization is important in general, but whether the specific interfaces and structural transitions revealed in this study are functionally and structurally relevant.

1. For examples, authors state that DnaT linker residues Asp66 and Asp70 are important for interactions with PriB. The essentiality of these residues have not been examined previously. I suggest that authors examine the importance of these residues for interaction with PriB and for cellular functions (complementation of the dnaT mutant cells). This is also related to my question #3 below

2. I am also unclear about how DnaTNTD and DnaTCTD are coordinated together with linker segment connecting them. Does DnaTNTD oligomerization rely on the oligomerization of DnaTCTD in the preprimosome? This could be examined by looking at the complex structures of a NTD oligomerization mutant and CTD oligomerization mutant.

3. Does DnaT linker also oligomerize in the final mature complex? Or are they structurally flexible?

4. Nomenclature. Thank you for rephrasing primosome for preprimosome.

Mok M, Marians KJ. The Escherichia coli preprimosome and DNA B helicase can form replication forks that move at the same rate. J Biol Chem. 1987 Dec 5;262(34):16644-54. PMID: 2824502.

In the above paper, preprimosome is defined as the primosome minus the DNA G protein (on page 16652), as I stated in my earlier comments. Therefore, the complex this study focuses on is more accurately “prepreprimosome” not the “prepreimosome”. I apologize for my imprecise statement in my comments.

The word “prepreprimosome” does appear in the literatures, but, in this paper, may be this can be replaced with “preprimosome” for simplicity, although I suggest the authors point out the above somewhere in the text.

With regard to the point #1, if there are any literatures describing these acidic amino acid mutants of DnaT, please cite and explain in the text. If not, I would anticipate some additional experiments, if that is possible.

With regard to the point #2, while I understand that additional mutational and structural analyses may be beyond the scope of the current study, the manuscript would benefit from a more clear description of the potential roles of DnaTCTD and DnaTNTD, as well as that of DnaT linker in the final “mature” complex, and speculation on how individual mutations would affect the structures.

Open Access This Peer Review File is licensed under a Creative Commons Attribution 4.0 International License, which permits use, sharing, adaptation, distribution and reproduction in any medium or format, as long as you give appropriate credit to the original author(s) and the source, provide a link to the Creative Commons license, and indicate if changes were made.
In cases where reviewers are anonymous, credit should be given to 'Anonymous Referee' and the source.
The images or other third party material in this Peer Review File are included in the article's Creative Commons license, unless indicated otherwise in a credit line to the material. If material is not included in the article's Creative Commons license and your intended use is not permitted by statutory regulation or exceeds the permitted use, you will need to obtain permission directly from the copyright holder.
To view a copy of this license, visit https://creativecommons.org/licenses/by/4.0/

REVIEWER COMMENTS

Reviewer #1 (Remarks to the Author):

In E. coli, the PriA, PriB and DnaT primosome restarts replication at abandoned DNA forks. In this manuscript, Ducos, Duckworth and colleagues report cryo-EM structures of the primosome. They identify two different assemblies on a stalled replication fork. One intermediate shows the recruitment of a single DnaT protomer. The second structure, which they describe as the mature primosome, contain multiple DnaT protomers arranged as a helical filament that wraps around single stranded DNA emanating from PriB. The authors note a loose structural similarity between DnaT and the bacterial replication initiator, DnaA. Multimeric protein filaments wrapping around single stranded DNA might play the same role during the ensuing helicase loading process, be it for replication initiation or fork restart. This is a nice piece of work that should be published after addressing the issues listed below.

1. Currently, the summary of the results constitutes ~35% of the Introduction. That could be shortened, in my opinion.

We thank the reviewer for this comment. We have condensed the results summary in the final paragraph introduction (it is now ~28% of the introduction).

2. The authors claim that the presence of multiple DnaT molecules on PriA-PriB constitutes the structure of the mature replisome. I feel the authors should elaborate on why DnaT multimerisation is required for maturation, at this stage of the results section. Otherwise, such a statement belongs to the Discussion.

We realized, after reading this comment and a related comment from reviewer 3, that we had not provided a full description of the literature showing that DnaT multimerization is required for replication restart. A much fuller description is now provided throughout the manuscript.

In the introduction, we have added the following statements: Multiple DnaT molecules can assemble within the preprimosome, with higher-order DnaT oligomers forming as a function of DnaT concentration in vitro[44], suggesting that DnaT oligomerizes within the preprimosome. Isolated $DnaT^{NTD}$ and $DnaT^{CTD}$ fragments self-associate in vitro, and sequence changes in the domains that impair DnaT oligomerization weaken DnaT ssDNA binding in vitro and block DnaT functions in vivo[37,41,47].

In the same section, we note: Based upon prior biochemical studies of DnaT, we posit that the monomeric and filamented DnaT preprimosome structures represent intermediate and mature forms of the complex.

We have also added to a sentence in the results/discussion to highlight the importance of $DnaT^{CTD}$ oligomerization: Together with prior biochemical analysis that defined a handoff where

DNA is shuttled from PriB to DnaT[36,38,48] and that $DnaT^{CTD}$/$DnaT^{CTD}$ interface residues are critical for oligomerization and replication restart[37,41], the preprimosome structures suggest that DnaT linker docking and oligomerization are key for transitioning the lagging strand to DnaT.

In the results/discussion, we have added: Mutation of key residues at the $DnaT^{NTD}$ dimer interface (V10, F35) and at the dimer-dimer interface (F42, Y43) that impede oligomerization leads to defects in vivo, showing the importance of $DnaT^{NTD}$ multimerization in replication restart[41,47,50].

3. The authors claim that DnaT binds DNA with opposite polarity compared to the crystal structure. How did they determine polarity? Is the nucleic acid resolution sufficient to substantiate this claim? Is it continuous density between single stranded DNA and the duplex segment, where major and minor grooves can be discriminated? In either case, to substantiate their claim, the authors should add a supplementary figure, with the model overlaid to the transparent map. This is especially important given that the atomic model presented here has inverted DNA binding polarity compared to a previous co-crystal structure.

We agree that the difference in ssDNA polarity between our structures and the previous DnaT C-terminal domain structure is an interesting and important point. As suggested, we have added a new supplementary figure 1 showing how the oligonucleotides anneal to form the fork, along with a comparison between our DnaT/ssDNA filament and the published crystal structure. This figure emphasizes the opposite polarity of the ssDNA within the DnaT protomers and the quality of both maps.

The polarity of the lagging strand in our PriA/PriB/DnaT/DNA structures is determined by the design of the synthetic replication fork. The 15 lagging-strand bases lack an annealing partner in the fork, while the leading and parental arms are double-stranded. In the structure, we see two distinct regions of double-stranded DNA along with a region of single-stranded DNA extending from the parental duplex. Therefore, the polarity of the single-stranded lagging DNA is defined by the overall design of the replication fork used in our complex, with the terminal nucleotide at the 5′ end of the DNA.

4. When drawing a parallel between DnaT and the DnaA initiator, the authors should point out that DnaA is a right-handed filament while DnaT is a left-handed filament and perhaps comment on the functional implications.

We thank the reviewer for highlighting this difference. We have included the following statement about the different handedness in the relevant discussion section:
Notably, while the $DnaT^{CTD}$ forms a left-handed nucleoprotein filament, the $DnaA^{CTD}$ filament is right-handed[4].

5. The figures could be improved by adding color code in every figure (space seems available) and pointing to specific features of note in the structure (e.g. ssDNA contact by PriB in Figure

1B for example). In Figure 1, I feel that purple DNA against highly shaded grey protein is barely visible, at least for me.
We thank the reviewer for their concern about the purple DNA being hard to see against the grey protein. Because it is difficult to color multiple species in different colors at various points in the paper, we experimented with several color schemes. However, although some options improved the DNA visibility against the grey background, they made other images worse.

We have now added a comment in the figure legends to describe the color scheme, referring to the first structure figure.

6. Figure 5. It would be useful for the authors to explain whether they think the DnaT filaments interspersed by PriB could be physiological or not. Also the figure could be improved. There are white arrows pointing at DnaT filaments but labels for PriB could be added.

As suggested we have added the following statement regarding the interspersing of PriA/PriB complexes on the DnaT^{NTD} filament:

These superstructures of multiple PriA/PriB assemblies bound to long DnaT^{NTD} filaments are likely due to the high relative concentration of the purified proteins in the sample and not physiologically relevant as only a single mature preprimosome would assemble per replication restart site.

We have also added arrows to Figure 5A to locate the multiple PriA/PriB/replication fork complexes along the DnaT^{NTD} filament.

Reviewer #2 (Remarks to the Author):

Ducos & Duckworth et al. used cryo-EM to determine structures of the E. coli primosome (PriA, PriB, DnaT), aiming to reveal the structural role of DnaT. They were able to determine two distinct forms of the complex, representing intermediate and mature primosomes. Based on these structures, they propose a structural model for the PriA/PriB/DnaT DNA replication restart pathway and a mechanism that shows parallels with replication initiation. The data are at high enough resolution to answer the questions.

This work will be of significance to the DNA replication and genome maintenance fields, with potential future use of this knowledge to target antimicrobial resistance.

Minor comments:

How were the synthetic replication fork lengths (25-base pair dsDNA parental arm, a 15-base pair dsDNA leading strand, and a 15-base ssDNA lagging strand) chosen?

The following description of the rationale behind replication fork arm lengths has been added to the methods section:

The synthetic DNA replication fork used in EM was generated as described previously (Table 1)[30]. The DNA lengths were chosen based on previous crosslinking and crystallography results. The PriA winged-helix domain contacts the parental duplex approximately 11-24 bp from the fork junction, thus we used a 25 bp parental duplex[28]. The PriA helicase domain contacts the lagging strand DNA approximately 5-6 nt from the fork junction[29]. This along with PriB's DNA binding footprint being around 10-15 nt lead us to choose a 15 nt long ssDNA lagging strand length[31]. PriA only interacts with the 3'-most nucleotide of the leading strand DNA[28]. Thus, we chose the leading strand to also be 15 bp to maintain high efficiency annealing.

The authors suggest that PriB is adapted so that one protomer binds to PriA and ssDNA while the second is available to bind the DnaT linker. Could the authors comment on the affinities of PriB with PriA, ssDNA, and DnaT, and whether those affinities would support such a mechanism?

We have added the following statement in the paper briefly describing this point:

The dual-binding capability of PriB is supported by previous binding studies showing that DNA increases the affinity of PriB for PriA and that PriB increases the affinity of DnaT for the PriA/DNA complex[36].

In the cell, the ssDNA lagging strand would be bound by SSB (perhaps even RecA). The authors should comment on how the presence of SSB could alter their model, or how SSB is displaced by the DnaT filament

We have added the following statement in the paper based on prior demonstration that PriA interaction with SSB modulates the SSB/ssDNA structure in a manner that exposes ssDNA:

If the lagging strand is duplex, PriA helicase activity catalyzes remodeling; if it is ssDNA, PriA modulation of ssDNA-binding protein/ssDNA complexes assembled on the lagging strand is used[25]. Exposure of lagging-strand ssDNA triggers movement of the PriA^{CRR}, allowing PriA to encircle the lagging strand.

Last paragraph of the introduction - the following sentence needs to be rewritten: Intriguingly, the positions of DnaT domains in the mature primosome model reveal an unexpected DnaA-like arrangement in which DnaTCTD filaments on ssDNA to mark the helicase-loading site, and with DnaTNTD element accessible for possible binding to DnaB/DnaC.

The final two sentences of the introduction have been edited to improve clarity:

Intriguingly, the positions of DnaT domains in the mature preprimosome model reveal a DnaA-like arrangement where the DnaT^{CTD} filaments on ssDNA to mark the helicase-loading

site and the $DnaT^{NTD}$ oligomerizes to potentially recruit the DnaB/DnaC complex. This parallel suggests that replication restart and canonical replication processes may share significant mechanistic similarities.

Figure 2 A, right – intermeditate = intermediate

We have fixed this error.

Reviewer #3 (Remarks to the Author):

The manuscript by Ducos et al reports the structures of PriA-PriB-DnaT assembly on a model stalled fork structure. They have identified two forms of assembly, one the authors call intermediate and the other mature. The authors propose a stepwise process for DnaT association in the complex; a single DnaT monomer first binds to PriA/PriB/DNA, followed by additional DnaT (DnaT-NTD) molecules extending a filament through oligomerization. They propose that DnaTCTD forms filaments on the ssDNA lagging strand, replacing PriB with DnaT. Based on the model, authors propose the similarity of DnaB/C complex recruitment between DnaA-OriC and PriA-stalled fork systems, but the model has not been experimentally tested.

The detailed structure of the bacterial fork restart primosome is an important issue and the authors have been conducting the structural studies of the factors required for the primosome. The current study deals with a protein assembly required for the helicase loading and represents an important step for complete understanding of fork restart mechanism.

My comments are as follows.

1. Comment on the title: Visualization of the complete primosome reveals the structural mechanisms governing DNA replication restart

Authors misunderstand the term “Primosome”. Primosome can lay down primer RNAs for initiation of lagging strand synthesis while unwinding the duplex DNA with its helicase activity. Therefore, Primosome (for the fork restart) includes PriA, PriB, (PriC), DnaT as well as DnaB helicase (these are called preprimosomal proteins) plus DnaG primase.

Authors need to correct the description throughout the manuscript. The structure discussed in this manuscript is not Primosome. Preprimosome is Primosome minus DnaG (which is isolatable and is associated with helicase activity due to inclusion of DnaB helicase). The structure they studied in this manuscript could be called “preprimosome”.

We thank you for pointing this out. We have updated the manuscript to reference “preprimosome” instead of “primosome”

2. It is of interest that two forms of assemblies were identified in the CryoEM. However, this

does not show that the intermediate is a prerequisite for the assembly of more “mature" complex. This needs to be verified by examination of the structures during time course assembly of the PriA-PriB-DnaT-complex. Alternatively authors need to show the transition from the intermediate to mature form in biochemical assays of “preprimosome” assembly.

We thank the reviewer for this comment. We realized, after reading this comment and a related comment from reviewer 1, that we had not provided a full description of the literature showing that DnaT multimerization is required for replication restart. A much fuller description is now provided throughout the manuscript.

In the introduction, we have added the following statements: Multiple DnaT molecules can assemble within the preprimosome, with higher-order DnaT oligomers forming as a function of DnaT concentration in vitro[44], suggesting that DnaT oligomerizes within the preprimosome. Isolated $DnaT^{NTD}$ and $DnaT^{CTD}$ fragments self-associate in vitro, and sequence changes in the domains that impair DnaT oligomerization weaken DnaT ssDNA binding in vitro and block DnaT functions in vivo[37,41,47].

In the same section, we note: Based upon prior biochemical studies of DnaT, we posit that the monomeric and filamented DnaT preprimosome structures represent intermediate and mature forms of the complex.

We have also added to a sentence in the results/discussion to highlight the importance of $DnaT^{CTD}$ oligomerization: Together with prior biochemical analysis that defined a handoff where DNA is shuttled from PriB to DnaT[36,38,48] and that $DnaT^{CTD}$/$DnaT^{CTD}$ interface residues are critical for oligomerization and replication restart[37,41], the preprimosome structures suggest that DnaT linker docking and oligomerization are key for transitioning the lagging strand to DnaT

In the results/discussion, we have added: Mutation of key residues at the $DnaT^{NTD}$ dimer interface (V10, F35) and at the dimer-dimer interface (F42, Y43) that impede oligomerization leads to defects in vivo, showing the importance of $DnaT^{NTD}$ multimerization in replication restart[41,47,50].

3. Authors need to verify the functional and biological significance of the structures identified.

We thank the reviewer for their comment. We have addressed the subpoints below with a more detailed description of the prior literature that highlights the significance of the interfaces in the new structures.

3-1 They propose the critical roles of DnaTNTD oligomerization. To demonstrate this model, authors need to identify the residues that are important for DnaT-NTD ologimerization, mutate them and examine their effects on preprimosome assembly in vitro as well as on in vivo functions. Similar studies need to be conducted for the DnaT-CTD oligomerization interaction surface to examine the functional importance of DnaT-CTD filamentation.

In our revised manuscript, we highlight prior work from Inoue et al. (2023, BBA-Proteins Proteomics, v. 1871, 140929), who characterized several DnaT^{NTD} variants *in vitro* and *in vivo*. Their paper examined residues responsible for the dimer interface (F42, Y43, L50, L53, L54) and dimer-of-dimer interface (V10, F35). Mutations of these residues caused defects in *in vitro* oligomerization assays and activity *in vivo*. These observations demonstrate that stability and oligomerization of the DnaT^{NTD} is important for its physiological role.

Similarly, McCool et al. (2004, Genetics, v. 167, p. 569-578) characterized the impact of a 6-residue deletion in DnaT^{CTD} (residues 87-92) *in vivo*. A mutant *E. coli* strain carrying this mutation was severely compromised, with high levels of SOS induction and low survival under UV stress. This region contains at least one residue (K88) that was suggested by Liu et al. (2014, Nucleic Acids Research, v. 42, p. 9470-9483) to be important for protomer-protomer interactions. Furthermore, Lui et al. showed that mutations in the DnaT^{CTD} disrupted DNA binding and filamentation *in vitro* and that DnaT variant proteins lacking the N-terminal region (DnaT$^{84\text{-}179}$) and lacking both the N- and C-terminal regions (DnaT$^{84\text{-}153}$) had lower cooperativity in a DNA binding assay.

We have added the following statements to the introduction to reflect these previous results:

Isolated DnaT^{NTD} and DnaT^{CTD} fragments self-associate in vitro, and sequence changes in the domains that impair DnaT oligomerization weaken DnaT ssDNA binding in vitro and block DnaT functions in vivo[37,41,47].

In the results/discussion, we have added: Mutation of key residues at the DnaT^{NTD} dimer interface (V10, F35) and at the dimer-dimer interface (F42, Y43) that impede oligomerization leads to defects in vivo, showing the importance of DnaT^{NTD} multimerization in replication restart[41,47,50].

We have also added to a sentence in the results/discussion to highlight the importance of DnaT^{CTD} oligomerization: Together with prior biochemical analysis that defined a handoff where DNA is shuttled from PriB to DnaT[36,38,48] and that DnaT^{CTD}/DnaT^{CTD} interface residues are critical for oligomerization and replication restart[37,41], the preprimosome structures suggest that DnaT linker docking and oligomerization are key for transitioning the lagging strand to DnaT

3-2 Is the linker of DnaT that authors claim binds to PriB important for the function of DnaT? What is the function of DnaT(∆linker) or DnaT mutant mutated in the PriB interacting amino acids (shown in Figure 3B) in the in vitro assays as well as in the cells?

Fujiyama et al. (2019, BBA – Proteins Proteomics, v. 1867, p. 367-375) identified several residues in the DnaT linker (60-88) that, when mutated, fail to compete with PriB for ssDNA binding. Several papers (Fujiyama et al. 2019, BBA – Proteins Proteomics, v. 1867, p. 367-375, Abe et al. 2021, FEBS Lett. 595(3):341-350) have identified PriB H26 as important for interaction with DnaT. While PriB H26A can bind ssDNA like WT PriB, it is defective in binding

DnaT$^{1-88}$, competing with DnaT$^{1-88}$ for ssDNA binding, and cannot complement the loss of *priB* function. These results show the importance of the DnaT linker competing with ssDNA for binding to the PriB basic grooves. Our models provide context for this competitive interaction.

We have highlighted these results in the revised manuscript by adding :

PriB variants that disrupt the DnaT linker docking site are non-functional in E. coli[45,46].

Reviewer #3 (Remarks to the Author):

The authors have revised the manuscript and have substantially improved the text by incorporating relevant biochemical literatures on DnaT oligomerization and function. These additions strengthen the plausibility of the proposed model for preprimosome maturation and clarify the basis for interpreting the filamented DnaT state as a functionally relevant assembly.

The current revision relies primarily on previously published mutational analyses of DnaT NTD and CTD oligomerization, rather than testing predictions that arise directly from the present structures.

Both DnaTNTD and DnaTCTD can self oligomeiize, and the authors state that oligomerization of the both domains is important. The mutations that inactivate the oligomerization activity of DnaT are reported to lose its functions.

However, I would like to emphasize that my original concern was not simply whether DnaT oligomerization is important in general, but whether the specific interfaces and structural transitions revealed in this study are functionally and structurally relevant.

1. For examples, authors state that DnaT linker residues Asp66 and Asp70 are important for interactions with PriB. The essentiality of these residues have not been examined previously. I suggest that authors examine the importance of these residues for interaction with PriB and for cellular functions (complementation of the dnaT mutant cells). This is also related to my question #3 below

> As noted in the papers (refs Abe et al (2021) FEBS Lett v. 595, p. 341-350 and Fujiyama et al (2019) BBA v. 1867, p. 367-375), the PriB side of the interface has been shown to be important in vivo and in vitro but the DnaT side of the interaction has only been probed in vitro. Our structure will guide future interrogation of this interface in vivo.

2. I am also unclear about how DnaTNTD and DnaTCTD are coordinated together with linker segment connecting them.

Does DnaTNTD oligomerization rely on the oligomerization of DnaTCTD in the preprimosome? This could be examined by looking at the complex structures of a NTD oligomerization mutant and CTD oligomerization mutant.

> As shown in prior publications cited in the paper, both the DnaT NTD (Inoue et al (2023) BBA Proteins and Proteomes v. 1871, 140929) and the DnaT CTD (Liu et al (2014) NAR v. 42, p. 9470-9483) are able to self-interact in isolation and sequence changes of the interfaces impact self-association and activity in vivo. We suspect that DNA-dependent oligomerization of the DnaTCTD increases local concentration of the DnaTNTD, stimulating NTD oligomerization. Additional structures with oligomerization variants could help to show that, as predicted by prior studies and the current structure, interface changes impact oligomerization.

3. Does DnaT linker also oligomerize in the final mature complex? Or are they structurally flexible?

> In a sense, this depends on where one defines the start and end of the linker sequence. We see density for predicted helices protruding from the NTD that may interact with one another, but not the rest of the linker. This suggests that the structured part of the linker can oligomerize, but the PriB-interacting part of the linker is structurally flexible and only one linker is observed in our EM maps.

4. Nomenclature. Thank you for rephrasing primosome for preprimosome.

Mok M, Marians KJ. The Escherichia coli preprimosome and DNA B helicase can form replication forks that move at the same rate. J Biol Chem. 1987 Dec 5;262(34):16644-54. PMID: 2824502.

In the above paper, preprimosome is defined as the primosome minus the DNA G protein (on page 16652), as I stated in my earlier comments. Therefore, the complex this study focuses on is more accurately “prepreprimosome” not the “prepreimosome”. I apologize for my imprecise statement in my comments.

The word “prepreprimosome” does appear in the literatures, but, in this paper, may be this can be replaced with “preprimosome” for simplicity, although I suggest the authors point out the above somewhere in the text.

> We agree that, for simplicity, “preprimosome” is most appropriate and is used throughout the manuscript to define the PriA/PriB/DnaT complex minus DnaB/DnaC/DnaG. In an attempt to avoid further confusion, we have also explicitly defined what we mean by preprimosome in the abstract and introduction sections of the manuscript.

With regard to the point #1, if there are any literatures describing these acidic amino acid mutants of DnaT, please cite and explain in the text. If not, I would anticipate some additional experiments, if that is possible.

> We have summarized all studies of which we are aware that look at the DnaT D66-E76 portion of the linker in the manuscript and in the response to point #1 above. Gel shift, NMR, and ITC experiments have been used to define the interaction between this portion of the DnaT linker and PriB. In vivo analysis has been done on PriB residue mutants but not on DnaT. We believe that these experiments are important but are out of the scope of our current manuscript. The structures we present here will be used to drive future experiments.

With regard to the point #2, while I understand that additional mutational and structural analyses may be beyond the scope of the current study, the manuscript would benefit from a more clear description of the potential roles of DnaTCTD and DnaTNTD, as well as that of DnaT linker in the final “mature” complex, and speculation on how individual mutations would affect the structures.

> We hypothesize that the DnaTCTD and DnaTNTD play analogous roles to the CTD and NTD of DnaA, respectively, while the role of the linker may be in coordinating the loading of the first DnaT in the PriA/PriB complex. This is discussed in the final section of our manuscript.
